# Correlation between Selected Clinical Symptoms and Severity of Aggression, Impulsiveness and Their Selected Behavioral Manifestations in Patients with Polycystic Ovary Syndrome Phenotype A

**DOI:** 10.3390/metabo13050646

**Published:** 2023-05-09

**Authors:** Aleksandra Barabasz-Gembczyk, Wojciech Mędrala, Patryk Rodek, Barbara Alli-Balogun, Jan Chrobak, Marlena Cwynar, Dominika Sikora, Mariusz Wójtowicz, Grzegorz Franik, Paweł Madej, Krzysztof Kucia

**Affiliations:** 1 Department and Clinic of Adult Psychiatry, Faculty of Medical Sciences, Medical University of Silesia in Katowice, Ziołowa 45/47, 40-635 Katowice, Polandbarbara.balogun@gmail.com (B.A.-B.);; 2 Department of Gynecological Endocrinology, School of Medicine in Katowice, Medical University of Silesia, 40-055 Katowice, Polandgfranik@sum.edu.pl (G.F.);; 3 Department of Gynecological and Obstetrics Women’s and Child Health Center, Medical University of Silesia, 41-803 Zabrze, Poland; 4 Infertility Outpatient Clinic, University Clinical Center, Medical University of Silesia, 40-042 Katowice, Poland

**Keywords:** alcohol consumption patterns, BMI, eating habits, PCOS, psychoneuroendocrinology, risky sexual behavior

## Abstract

Previous studies on aggressiveness and impulsiveness in women with polycystic ovary syndrome (PCOS) are ambiguous. Furthermore, no biochemical or clinical factors related to these variables have been definitively confirmed. The aim of the study was to clarify whether, in women with phenotype A of PCOS, variables such as body mass index and clinical and biochemical hyperandrogenism have an impact on either the intensity of impulsivity or aggression or on other selected behavioral manifestations of these variables. The study included 95 patients diagnosed with PCOS phenotype A. The criterion for recruitment into the study group and the control group was body mass index. The study was conducted with the use of a closed-format questionnaire and calibrated clinical scales. Higher body mass index (BMI) values in women with PCOS phenotype A are associated with poor eating habits. The severity of impulsivity and aggression syndrome, as well as the tendency to engage in risky sexual behavior and patterns of alcohol consumption among patients diagnosed with PCOS phenotype A, are not dependent on BMI. The severity of impulsiveness and the syndrome of aggression in women with phenotype A PCOS are not associated with clinical symptoms of hyperandrogenism or with androgen levels.

## 1. Introduction

PCOS is a complex endocrine and metabolic disorder, affecting, depending on accepted diagnostic criteria, anywhere from 6% to 13% of women of reproductive age [1]. PCOS is associated with symptoms such as ovulatory disorders, hyperandrogenemia, clinical hyperandrogenism, impaired secretion of gonadotropic hormones, insulin resistance, obesity, infertility, and lipid and carbohydrate metabolism disorders [2]. Increasing attention has been attributed to the co-occurrence of eating disorders and abnormal eating habits in women with PCOS [2,3]. Among the factors predisposing to this coincidence, the literature cites increased body mass index (BMI), greater dietary restrictions, dissatisfaction with one’s own body, low self-esteem, co-occurrence of depressive symptoms, anxiety, distress, and poorer quality of life [4,5].

The neurobiological basis of impulsive and aggressive behavior has not been precisely defined due to the complexity of interactions between biological and environmental factors [6]. Testosterone and serotonin have been linked to high levels of aggression and impulsivity for years [7]. Studies show a high correlation between testosterone levels and aggressive behavior and feelings of hostility and anger in both men and women [8]. 

Obesity can be viewed as an expression of emotional urges, habitual impulsivity, and self-aggression [9]. In addition, effective inhibition of responses to sexual stimuli has been shown to be inversely related to overall impulsivity [10]. Moreover, high impulsivity is associated with a higher frequency of engaging in risky and compulsive sexual behavior, hypersexuality, low levels of sexual restraint, and a tendency toward infidelity [10]. Recent studies indicate that alcohol-dependent individuals are characterized by greater impulsivity [11]. High behavioral impulsivity is also associated with a worse course of addiction, while cognitive impulsivity leads to an early onset of drinking [12]. Based on these reports, we created the following research hypotheses: Firstly, there are differences in the severity of impulsivity and aggression syndrome and their selected behavioral aspects among women with phenotype A PCOS dependent on their BMI, severity of symptoms of clinical hyperandrogenism, and results of biochemical tests. Secondly, there are different relationships between the dominant direction and dimensions of aggressiveness and impulsivity and selected behavioral aspects, i.e., incorrect eating habits, risky sexual behavior, and alcohol consumption patterns, among women with PCOS phenotype A depending on their BMI, severity of symptoms of clinical hyperandrogenism, and results of biochemical tests.

## 2. Materials and Test Methods

**Figure 1:**
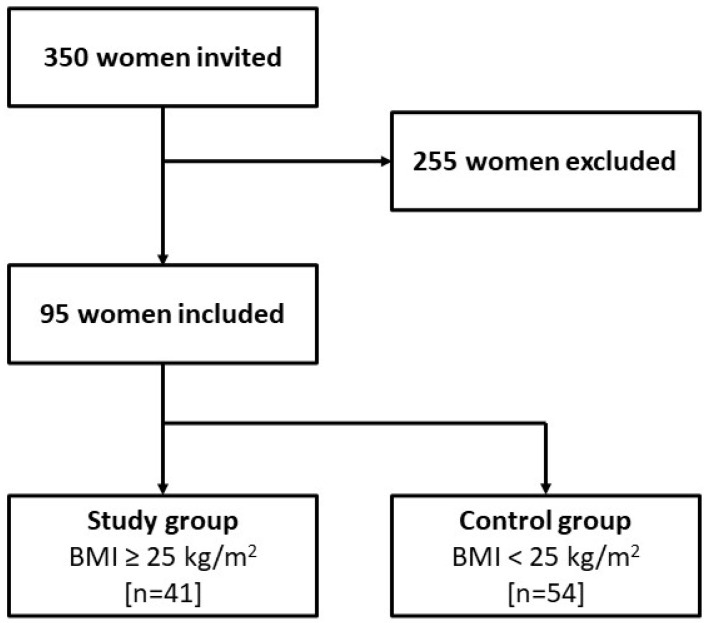
Flow diagram for inclusion and exclusion in the study and control groups.

The study enrolled 95 women with PCOS phenotype A, diagnosed according to the Rotterdam criteria, who were hospitalized in the Department of Gynecological Endocrinology of the Prof. Kornel Gibinski University Clinical Center in Katowice, Poland. Information from the department’s medical records, including the diagnosis of PCOS phenotype A, biochemical test results, assessment of hyperandrogenism symptoms, measurement of anthropometric parameters, and demographic data, was used in the study. Initially, 350 women were invited to take part in the study; 255 participants were excluded from the final analysis as they did not meet all inclusion criteria and/or due to incompletely completed questionnaires. A total of 95 participants were qualified for the study, of whom 41 patients (43.16%) with a BMI ≥ 25 kg/m^2^ were included in the study group. The remaining 54 patients (56.84%) with a BMI below 25 kg/m^2^ were included in the control group (Figure 1). 

The mean age values were 26.05 years for the study group and 25.24 years for the control group. In both the study group and the control group, the youngest person was 19 and the oldest was 34 (Table 1). 

Inclusion criteria for the study and control group were: (1) age above 18 years and below 35 years; (2) diagnosis of PCOS phenotype A according to the Rotterdam criteria; and (3) informed consent to participate in the study. Exclusion criteria for the study and control group were: (1) diagnosis of psychiatric diseases and disorders, including psychoactive substance use disorders (excluding caffeine and nicotine); (2) use of hormonal medications or medications affecting hormone levels and dietary supplements in the last 6 months; (3) diagnosed endocrine and metabolic diseases other than PCOS (e.g., thyroid disease, adrenal disease, diabetes mellitus); (4) incomplete completion of the study questionnaire; and (5) lack of consent to participate in the study. 

All patients were informed of the principles and purpose of the study and qualified for it after obtaining informed consent. The study was carried out with care for the patients’ anonymity; the self-completed sheets were returned in a folder attached to the questionnaires to ensure respondents’ anonymity. 

PCOS was diagnosed on the basis of the occurrence of three Rotterdam criteria. A routine ultrasound examination of the ovaries was performed by an experienced ultrasonographer using the Voluson 730 Expert device after a gynecological examination and assessment of androgen serum levels of ovarian and adrenal origin. In all patients who fasted, in the morning, anthropometric parameters such as BMI and height were measured. Body weight was assessed with the use of a certified electronic scale with an accuracy of 0.1 kg and height with an accuracy of 0.5 cm. A BMI nutrition assessment was made according to WHO criteria [13]. Blood samples were taken in all patients in the morning, 16 h after the last meal, in the follicular phase between the 2nd and 5th days of the menstrual cycle. Subsequently, the levels of androgens, total testosterone (TT), free testosterone (FT), and sex hormone-binding globulin (SHBG) were tested. The free androgen index (FAI) was calculated from the formula: FAI = (TT/SHBG) × l00%.

Standardized scales and self-completion inventories were used to assess the severity of individual variables: the Barratt Impulsiveness Scale (BIS-11—Barratt Impulsiveness Scale version 11), the Psychological Inventory of Aggression Syndrome (I.P.S.A.) by Professor Zbigniew Gaś, the Eating Related Behaviours Questionnaire (KZZJ) by Professor Nina Ogińska-Bulik, the Alcohol Use Disorders Identification Test (AUDIT), and the Sexual Risky Survey (SRS) by Turchik & Garske, 2009 [9,14,15,16,17]. The research was conducted between November 2018 and October 2020. The research project was assessed in accordance with the resolution of the Bioethics Committee of the Silesian Medical University in Katowice (No. KNW/0022/KB/243/18 of 7 November 2018) as not having the character of a medical experiment in light of the Act of 5 December 1996 on the professions of physician and dentist (Journal of Laws of 2018, item 617, as amended) and not requiring the assessment of the Bioethics Committee.

## 3. Statistical Analysis

The collected data were coded and transferred to a database prepared in the Statistica 13.3 package. Statistical analysis of the data included elements of descriptive and analytical statistics, and a *p*-value < 0.05 was used as the criterion for statistical significance.

To describe the quantitative variables, arbitrary measures of central tendency (mean, median, and quartiles) were used along with measures of dispersion (standard deviation, or interquartile distance—IQR), and the Shapiro-Wilk test was used to assess the normality of the distribution of variables identifying the assessed domains of the individual questionnaires. For the interpretation of the variation of quantitative variables in independent groups defined by their membership in the study group or control group as well as by their pattern of alcohol consumption, non-parametric tests (the Mann-Whitney U test and the Anova Kruskal-Wallis test) were applied due to the deviation of the distribution of variables from the normal distribution. As a countermeasure, due to the small size of the study and control groups (as well as the deviation of the variable distributions from the normal distribution), the evaluation of the significance and direction of the relationships between the individual quantitative variables was carried out using Spearman’s r correlation. 

## 4. Results

### 4.1. Severity of Impulsivity

There were no statistically significant differences in scores for all analyzed variables between the study group and the control group (Table 2).

### 4.2. Severity of Aggression

There were no statistically significant differences in scores for all analyzed variables between the study group and the control group (Table 3).

### 4.3. Eating Habits

The statistical analysis of the following factors: total eating habits, emotional overeating, and dietary restrictions showed statistical significance (*p* < 0.001, *p* = 0.003, and *p* < 0.001, respectively) (Table 4).

### 4.4. Predisposition to Engage in Risky Sexual Behavior

There were no statistically significant differences in scores for the assessed factors of the SRS questionnaire between the study group and the control group (Table 5).

### 4.5. Patterns of Alcohol Consumption 

**Figure 2:**
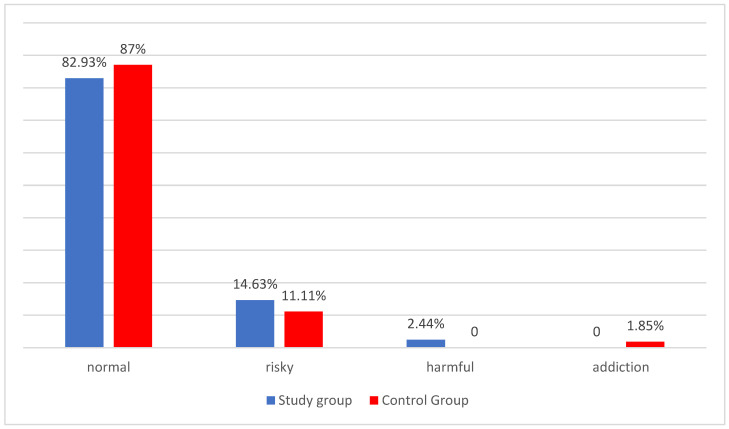
Patterns of alcohol consumption in the study group and the control group.

It was observed that the risky pattern of alcohol consumption was slightly more frequent in the study group (14.63%; N = 6) than in the control group (11.11%; N = 6), while the low-risk drinking pattern was more frequent in the control group (87.04%; N = 47) versus in the study group (82.93%; N = 34) (Figure 2). 

### 4.6. Correlation between Impulsivity and Severity of Clinical Hyperandrogenism Symptoms and Biochemical Test Results 

There were no statistically significant differences in scores for the assessed factors of clinical hyperandrogenism symptoms and biochemical test results (Table 6).

### 4.7. Correlation between Aggression and Severity of Clinical Hyperandrogenism Symptoms and Biochemical Test Results

There were no statistically significant differences between aggression and severity of clinical hyperandrogenism symptoms and biochemical test results (Table 7).

## 5. Discussion

As expected, the study indicated an increase in abnormal eating habits in the group of women with a BMI ≥ 25 kg/m^2^ compared to the normal-weight group among patients with PCOS phenotype A. According to the literature review, the prevalence of eating disorders, especially bulimia and binge eating disorders, as well as the presence of unhealthy eating habits, is higher in women with PCOS compared to healthy women, as suggested by some researchers, irrespective of their BMI [18,19,20,21,22]. No publications evaluating differences in the severity of abnormal eating habits between the normal-weight group and the group of women with a BMI ≥ 25 kg/m^2^ and PCOS phenotype A were found in the available literature. The most similar study is the one published in 2019 by Pirotta et al. The study showed that abnormal eating habits are more common in women with PCOS compared to a group of healthy women, and a dietary restriction is the only abnormal eating habit that occurs more frequently in the group of women with PCOS. They also emphasized that a significant predictor of the occurrence of both eating disorders and poor eating habits is increased BMI alone, irrespective of PCOS [23].

The study showed no significant differences in the severity of impulsivity and aggression between the normal-weight group and the group of women with a BMI ≥ 25 kg/m^2^ and PCOS phenotype A. Unfortunately, the results obtained cannot be compared with those of other authors, as no available publication on aggression and impulsivity in women with PCOS has divided the control and study groups according to BMI in women with PCOS phenotype A. In all available studies addressing aggression in women with PCOS, the control group was composed of healthy women. Researchers report, among other things, a higher number of hospitalizations for self-harm (7.2% to 2.9%), more frequent suicide attempts (14% to 2%), and more intense feelings and expressions of anger combined with poorer self-control in women with PCOS compared to healthy women [24,25,26]. In addition, the available studies on the severity of anger expression in relation to BMI only apply to women without PCOS and show no significant correlation between those variables [27]. Most of the available literature focuses on the study of the severity of impulsivity as one of the symptoms of psychiatric disorders such as Attention Deficit Hyperactivity Disorder or impulse control disorders observed in eating disorders, the prevalence of which is described as higher in a group of women with PCOS compared to women without PCOS [28,29]. 

The study further showed that there were no statistically significant differences in scores for the assessed scales of the SRS questionnaire between the study group and the control group. In the available literature, there are both descriptions of the prevalence of sexual dysfunction in patients with PCOS compared to groups of women without the syndrome and the absence of such differences [30,31,32,33,34,35,36,37,38,39]. In addition, the relationship between BMI and sexual functioning in women with PCOS is inconclusive [40]. Studies comparing the sexual functioning of obese and normal-weight PCOS patients provide the following findings: higher BMI is associated with a significant reduction in orgasm, reduced sexual activity, reduced sexual satisfaction, overall poorer sexual functioning, and a sense of lower sexual attractiveness [30,31,35,40,41]. Interestingly, it is noted that obesity and overweight alone do not affect sexual activity, but the co-occurrence of obesity and PCOS may interfere with it [40]. No publications were found in the available literature assessing the difference in severity of the tendency to engage in risky sexual behavior among women with PCOS phenotype A with a BMI-based stratification.

In the next stage of the study, it was observed that the risky pattern of alcohol consumption was slightly more frequent in the study group than in the control group, while the low-risk drinking pattern was more frequent in the control group. Given the very high prevalence of individuals in the overall population included in the analysis whose pattern is defined as a low-risk pattern (81 women), which represents a deviation of the distribution of the variables from the normal distribution for the interpretation of the variation in these quantitative variables, a non-parametric test (Anova Kruskal-Wallis) was used. Considering that more caution should be exercised with this test in drawing far-reaching conclusions from the study, it was decided to terminate the study of the severity of these characteristics at this stage, making it impossible to demonstrate the statistical significance of these results. There is a clear gap in the available literature when it comes to research on alcohol consumption patterns in women with PCOS. In the Chinese study published in 2014, Zhang J. et al. described alcohol drinking as an independent risk factor for PCOS [42]. In contrast, Zhang B. et al. in 2020 found no difference between the three study groups (a group of women with PCOS and oligo-ovulation, a group of women with PCOS and normal ovulation, and a group of healthy women without PCOS) in alcohol consumption patterns [43]. Similar findings were published by Sánchez-Ferrer et al. in 2020 and Cutillas-Tolín et al. in 2021, showing no differences in alcohol consumption patterns between a group of women with PCOS and a group of healthy women [44,45]. However, all the cited studies differ significantly in methodology, did not use the AUDIT test, and only asked about drinking alcohol. No studies comparing alcohol consumption patterns in women with PCOS phenotype A with a BMI-based stratification were found in the available literature.

In both the study group and the control group, there were no statistically significant correlations between the severity of impulsivity and the symptoms of clinical hyperandrogenism or the results of biochemical tests. There are inconclusive reports in the literature on the relationship between testosterone levels and impulsivity in healthy women without PCOS, confirming an indirect effect or showing a negative correlation [46,47]. An association has been shown between higher testosterone concentrations and more severe symptoms of Attention Deficit Hyperactivity Disorder in women without diagnosed PCOS [48]. Özdil et al. found a significant correlation between increased levels of total testosterone (TT) and higher scores on the total impulsivity scale, as well as between increasing values of the free androgen index (FAI) and higher scores on the total impulsivity scale, or impulsivity due to a lack of planning and motor impulsivity [49]. In addition, they showed a significant correlation between decreasing levels of sex hormone-binding globulin and the motor impulsivity scale and impulsivity related to a lack of planning. No such relationship has been demonstrated in this study. It is difficult to explain the reasons for this discrepancy given that the study populations were very similar in terms of both age and size, the location of the study, and the fact that the same questionnaire was used in both studies to assess impulsivity. A noticeable difference is the selection of the group. It is possible that the final results were influenced by ethnic, cultural, and genetic differences between the groups of women included in the study. The study by Özdil et al. was conducted in Turkey, while this one was carried out in Poland.

No statistically significant associations were found between indicators of aggression severity, symptoms of clinical hyperandrogenism, and biochemical test results in the compared groups. There are descriptions in the available literature of a relationship between testosterone concentrations and the severity of aggression and anger, but the available studies differ significantly in the population of subjects [7,8,50]. Barry et al. published a study negating the relationship between androgen levels and aggression severity in women with PCOS; however, in their study, the control group was infertile women without PCOS [51]. Again, the only findings with which to compare the results obtained in this study are those of a previously cited study published by Özdil et al. The study showed that as TT and FAI concentrations increase, the feeling and expression of anger also increase, and the ability to control and inhibit its expression decreases. They identify FAI as a predictor of feeling, expressing, and controlling anger [49]. In the present study, a complementary relationship was not obtained. As before, it is difficult to explain the reasons for this discrepancy given that the study populations were very similar in terms of age, size, and location. Different questionnaires assessing the severity of aggression or anger were used as in the previous case. It is also possible that the final results were influenced by ethnic, cultural, and genetic differences between the groups of women included in the study.

Several clinical implications of our results need to be considered. Firstly, a thorough medical interview with an assessment of eating habits should be conducted for patients with PCOS. Finding people with poor eating habits would allow them to receive additional psychological, dietary, or psychiatric support. Secondly, detailed psychoeducation in the field of healthy lifestyles should be provided. Finally, the results of this work could serve to determine the areas for further investigation of the mechanisms and severity of impulsivity and aggression in patients with PCOS, with special attention to their behavioral manifestations.

There are various limitations to the study that need to be considered. Firstly, we did not run a power analysis to assess the adequate sample size prior to the enrollment of patients, which is a major limitation to our study. The sample size of the study group was relatively small, which might contribute to false positive or false negative results; thus, replication studies with larger sample sizes are necessary. This is due to the broad exclusion criteria, which resulted in a 56% reduction in the study population. Secondly, the study design, based on self-administered questionnaires, retrospectively resulted in a negative impact on the sample size. A large number of the respondents withdrew from participating in the survey when filling out the questionnaires (16% of the respondents). Furthermore, we cautiously speculate that some of the questions, especially those related to alcohol consumption patterns or engaging in risky sexual behavior after psychoactive substance use, were answered with a certain degree of self-correction, driven by ongoing hospitalization and possible concern that such information might affect respondents’ treatment or influence the direction of further diagnosis. As with the behavioral manifestations of aggression and impulsivity in this population, there is relatively little data on the aggressiveness and impulsiveness of women with PCOS. In particular, the areas of risky sexual behavior, alcohol consumption patterns, and inappropriate eating habits in women with PCOS should be thoroughly assessed in the future. Such a scarcity of knowledge is reflected in the small number of sources that were cited in our study, especially those from the last five years. Consequently, further studies with larger groups are needed to confirm our findings.

## 6. Conclusions

Higher BMI values in women with PCOS phenotype A are associated with poor eating habits. 

The severity of impulsivity and aggression syndrome, as well as the tendency to engage in risky sexual behavior and patterns of alcohol consumption, among patients diagnosed with PCOS phenotype A are not dependent on BMI.

The severity of impulsiveness and the syndrome of aggression in women with PCOS phenotype A are not associated with clinical symptoms of hyperandrogenism or with androgen levels. 

## Figures and Tables

**Table 1:** Descriptive statistics of age and BMI in the study and control groups.

		Study Group	Control Group
N		41	54
Age	M ± SD	26.05 ± 4.11	25.24 ± 4.22
	Me ± IQR	25.00 ± 6.00	25.00 ± 7.00
	Min	19.00	19.00
	Max	34.00	34.00
	Q1/4	23.00	21.00
	Q3/4	29.00	28.00
BMI	M ± SD	32.30 ± 4.50	20.94 ± 2.16
	Me ± IQR	31.62 ± 5.90	20.89 ± 3.19
	Min	25.10	15.63
	Max	47.66	24.88
	Q1/4	28.48	19.53
	Q3/4	34.38	22.72

M—mean; SD—standard deviation; Me—median; IQR—interquartile range; Min—minimum; Max—maximum; Q1/4—lower quartile; Q3/4—upper quartile.

**Table 2:** Results of the Mann-Whitney U test for significance of differences in scores for BIS-11 dimensions between the study group and the control group.

BIS-11 Dimensions	Significance of Differences	
	Z	*p*
IC	−0.706	0.479
IU	−0.236	0.811
M	−1.840	0.064
IP	−0.612	0.053

IC—total impulsivity; IU—impulsivity of attention; IM—motor impulsivity; IP—impulsivity resulting from lack of planning; Z—outwardly directed aggression index; *p*—*p*-value.

**Table 3:** Results of the Mann-Whitney U test for significance of differences in scores for the I.P.S.A. indicators between the study group and the control group.

I.P.S.A. Indicators	Significance of Differences	
	Z	*p*
WO	0.139	0.889
O	0.398	0.690
S	0.695	0.487
U	−0.522	0.601
Z	0.927	0.353
K	−1.239	0.215

WO—general index of the aggression syndrome; O—propensity to retaliate; S—self-aggression index; U—latent aggression index; Z—outwardly directed aggression index; K—control of aggressive behavior; *p*—*p*-value.

**Table 4:** Results of the Mann-Whitney U test of significance for the Mann-Whitney differences in the scores for the KZZJ factors between the study group and the control group.

KZZJ Factors	Significances of Differences	
	Z	*p*
NŻ	−3.610	<0.001
NP	−1.735	0.083
EP	−2.915	0.003
RD	−3.745	<0.001

NŻ—total eating habits; NP—habitual overeating; EP—emotional overeating; RD—dietary restrictions; Z—outwardly directed aggression index; *p*—*p*-value.

**Table 5:** Results of the Mann-Whitney U test for significance of differences in scores for SRS factors between the study group and the control group.

SRS Factors	Significance of Differences	
	Z	*p*
SRS-T	−0.417	0.676
F1	0.086	0.931
F2	−0.751	0.452
F3	1.446	0.148
F4	0.165	0.868
F5	0.296	0.766

SRS-T—total risky sexual behavior; F1—sexual behavior with casual partners; F2—risky sexual intercourse; F3—impulsive sexual behavior; F4—intentions to engage in risky sexual behavior; F5—risky anal sexual intercourse; Z—outwardly directed aggression index; *p*—*p*-value.

**Table 6:** Spearman’s r correlation analysis between BIS-11 dimensions and severity of clinical hyperandrogenism symptoms and biochemical test results in the study group and the control group.

Study Group								
Variable	BIS-11 Dimensions							
	IC		IU		IM		IP	
	r	*p*	r	*p*	r	*p*	r	*p*
TT*	−0.22	0.167	0.03	0.858	−0.15	0.341	−0.24	0.131
FT	−0.10	0.526	0.00	0.998	−0.10	0.519	−0.10	0.510
FAI	−0.13	0.417	−0.15	0.350	−0.07	0.656	−0.06	0.711
mFG	0.00	0.955	−0.27	0.084	0.25	0.114	−0.02	0.879
SHBG	−0.02	0.880	0.20	0.212	−0.01	0.946	−0.13	0.421
**Control Group**								
**Variable**	**BIS-11 Dimensions**							
	**IC**		**IU**		**IM**		**IP**	
	**r**	***p***	**r**	***p***	**r**	***p***	**r**	***p***
TT*	0.02	0.860	0.17	0.228	−0.08	0.539	−0.06	0.666
FT	−0.07	0.628	0.03	0.844	−0.09	0.536	−0.20	0.133
FAI	0.00	0.996	−0.10	0.482	−0.04	0.791	−0.13	0.340
mFG	−0.13	0.361	−0.08	0.565	−0.03	0.837	−0.05	0.692
SHBG	−0.04	0.792	0.24	0.073	−0.10	0.490	0.07	0.605

IC—total impulsivity; IU—attention impulsivity; IM—motor impulsivity; IP—impulsivity due to lack of planning; TT*—TT (ng/mL); *p*—*p*-value; r—Spearman’s r correlation; mFG—modified Ferriman-Gallwey scores; FAI—free androgen index; SHBG—sex hormone-binding globulin.

**Table 7:** Spearman’s r correlation analysis between I.P.S.A. indicators and severity of clinical hyperandrogenism symptoms and results of biochemical tests in the study group and the control group.

Study Group												
Variable	I.P.S.A. Indicators											
	WO		O		S		U		Z		K	
	r	*p*	r	*p*	r	*p*	r	*p*	r	*p*	r	*p*
TT*	−0.01	0.956	−0.08	0.606	0.06	0.664	−0.09	0.575	0.11	0.496	−0.20	0.206
FT	−0.01	0.928	0.17	0.285	0.02	0.858	−0.04	0.770	0.02	0.887	−0.24	0.115
FAI	−0.17	0.278	0.00	0.996	−0.23	0.135	−0.19	0.233	−0.16	0.310	0.10	0.522
mFG	−0.02	0.873	0.08	0.582	−0.06	0.680	−0.02	0.915	−0.09	0.570	0.13	0.386
SHBG	0.23	0.145	0.02	0.909	0.29	0.064	0.23	0.132	0.28	0.077	−0.26	0.104
**Control Group**												
**Variable**	**I.P.S.A. Indicators**											
	**WO**		**O**		**S**		**U**		**Z**		**K**	
	**r**	***p***	**r**	***p***	**r**	***p***	**r**	***p***	**r**	***p***	**r**	***p***
TT*	0.10	0.468	0.08	0.549	0.13	0.328	0.15	0.291	0.03	0.805	−0.16	0.233
FT	0.01	0.900	−0.09	0.477	0.24	0.076	0.12	0.359	−0.11	0.426	−0.15	0.277
FAI	0.13	0.333	−0.03	0.845	0.21	0.128	0.11	0.427	0.01	0.916	−0.17	0.229
mFG	−0.07	0.584	0.05	0.685	−0.03	0.836	−0.09	0.497	−0.12	0.378	−0.08	0.526
SHBG	−0.09	0.483	0.10	0.437	−0.21	0.133	−0.03	0.808	0.01	0.927	0.03	0.819

WO—general index of aggression syndrome; O—retaliatory tendencies; S—self-aggression; U—latent aggression; Z—outwardly directed aggression; K—control of aggressive behavior; TT*—TT (ng/mL); *p*—*p*-value; r—Spearman’s r correlation; mFG—modified Ferriman-Gallwey scores; FAI—free androgen index; SHBG—sex hormone-binding globulin.

## Data Availability

Data is contained within the article or Supplementary Material (File S1).

## Supplementary Materials

The following supporting information can be downloaded at: https://www.mdpi.com/article/10.3390/metabo13050646/s1, File S1: Raw data.
